# A Shift in Time Saves ……

**DOI:** 10.5005/jp-journals-10071-23129

**Published:** 2019-03

**Authors:** Shrikanth Srinivasan

**Affiliations:** 1 Department of Critical Care Medicine, Manipal Hospital, Dwarka, New Delhi, India.

## Abstract

**How to cite this article:** Srinivasan S. A Shift in Time Saves ……. Indian J Crit Care Med 2019;23(3):109-110.

Late transfer out of the patients from the ICU to the wards after the routine hospital working hours (after-hours discharge) is a common and ever-increasing phenomenon in Indian ICUs. Its only in recent years that the possible relationship between time of discharge from ICU and hospital mortality has been recognized. Several studies have shown that after-hours discharge to ward could be associated with increasing risk of adverse outcomes including greater in-hospital mortality^1,2^, a higher unplanned ICU readmission rate^3,4^ and a prolonged hospitalization^4^.

In this issue, Chatterjee et al.^5^ in their single centre retrospective analysis of 1356 patients from a tertiary hospital in India have analysed the outcome of patients shifted during routine hours (8AM to 8 PM) (71.8% of patients) versus those shifted in after-hours (8 PM to 8 AM) (28.2%). This is the first study from India to study association of ICU transfer time and outcome.

They found that the unadjusted hospital mortality rate for after-hour-transfers was significantly higher compared to day- time-transfers (7.1% vs. 4.1%; *p* = 0.02). This finding persisted after adjustment for illness severity, wherein the after-hour-transfers were still associated with significantly higher hospital mortality compared to day-time-transfers (aOR1.7, 95%CI 1.1,2.8; *p* = 0.04). The median duration of hospital LOS and ICU readmission rate though higher for after-hour-transfers, was not statistically significant in adjusted analysis.

In their study, a wide mix of medical and surgical patients were evaluated and they found that a higher proportion of patients with elective admission to ICU (postoperative patients) were transferred out timely in comparison to medical patients who were sicker (having sepsis, needing higher requirement of vasopressors and mechanical ventilation) and had a higher APACHE IV scores.

This is a common phenomenon in our ICUs wherein patients who are admitted for observation after a planned procedure have a relatively more well planned, timely discharge from ICU compared to sicker, medical patients.

The authors in their study have highlighted the real and clear danger associated with after-hour-transfer. The issues being faced by their center include lack of availability of ward beds due to delayed discharge of patients from wards, poor staffing and surveillance during the after-hours and less access to HDU beds. These are common problems that are faced by most of the hospitals in India.

Several reasons have been cited for the delayed shifting from ICU including organizational aspects such as unavailability of a bed in the ward until late hours. This problem is common in our country where due to insurance related issues, the ward beds are not vacated on time leading to delayed transfer. Also, there is a pressure for additional admissions in ICU which at times can lead to premature transfers. Both premature discharge and delayed discharge is more likely to occur at night and each 1-hour delay is estimated to be associated with an adjusted 3% increase in the risk of mortality^6,7^. Delayed discharge may also increase the risk of ICU-acquired infections and psychological issues which independently influence post-ICU mortality and lead to additional delayed discharges. Other reasons could be poor discharge planning, financial constraints, unawareness of the risks posed by late discharge and non-availability of staff in wards.

Discharge from ICU is a crucial transition period, given that a patient who is recovering from a critical illness is being transferred to an area which has relatively less surveillance and is invariably understaffed with a lower doctor and nurse-to-patient ratio. The ward areas also have lesser monitoring devices and immediate access to life sustaining devices. The effects of this transition are compounded if patients are received in these areas after-hours.^6,7^

However, despite after-hours ICU discharge intuitively not being considered as optimal care, the evidence regarding a direct correlation between after-hour-transfer and poor outcome has been inconsistent and inconclusive. Several retrospective studies done in the western world have shown that there could be a correlation between after-hour-transfer and adverse outcomes^1- 4, 8 -10^. However, several other studies, including the more recent large- scale prospective study, failed to draw similar conclusions^11,12^. It is interesting to note that these studies were conducted in centers where there were enough ICU beds and there was no pressure for premature shifting and also that adequate staffing was available in ward areas, quiet unlike the conditions found in most Indian hospitals.

The discrepancies amongst the studies are likely influenced by the local healthcare systems, patient population, definitions of night time or weekend discharge, disease severity at admission or discharge, therapy limitations, sample size and study design.

Based on a comprehensive literature search, the newly revised American Society of Critical Care Medicine ICU practice guidelines recommend avoiding night time ICU discharge but these practical recommendations were graded as evidence level 2C, because they were formulated using a consensus review of contradictory research evidence^13^.

A recent meta-analysis^14^ on time to discharge and week end discharge on mortality has concluded that night-time-ICU discharge is associated with an increased risk of hospital mortality, while weekend ICU discharge is not. Given the methodological limitations and heterogeneity among the included studies, these conclusions should be interpreted with caution and should be tested in further studies.

On the whole latest shifting should be avoided as far as possible. High risk groups should be identified (based on age, comorbidities, severity of illness and disabilities) and all effort should be made to transfer these patients in a timely and careful manner.

Discharge planning needs to be done early and steps need to be taken by hospital organization that timely discharge from the ward is ensured so that the ICU patients could be shifted out within reasonable hours.

Also, it makes a statement for having more HDU beds as 64% of major adverse events that occur within 72 h after ICU discharge may be predictedandprevented by monitoringthe patient's vital signs^15^. Transfer to a ward is associated with increased post-ICU mortality, while transfer to a high dependency unit is not, which suggests that patients' outcomes may be associated with the intensity of post ICU medical care ^1^.

Late shifting from ICU is a phenomenon that is largely ignored because of both unawareness of its potential harm and lack of planning and resources. The implications, however, can be profound and potentially reverse all the efforts made to stabilize and shift the patient out in the first place and hence all efforts should be made to avoid after-hour-transfer.
